# DAEMDA: A Method with Dual-Channel Attention Encoding for miRNA–Disease Association Prediction

**DOI:** 10.3390/biom13101514

**Published:** 2023-10-12

**Authors:** Benzhi Dong, Weidong Sun, Dali Xu, Guohua Wang, Tianjiao Zhang

**Affiliations:** College of Computer and Control Engineering, Northeast Forestry University, Harbin 150040, China; nefudbz@nefu.edu.cn (B.D.);

**Keywords:** miRNA–disease association prediction, transformer, graph encoding, graph attention network

## Abstract

A growing number of studies have shown that aberrant microRNA (miRNA) expression is closely associated with the evolution and development of various complex human diseases. These key biomarkers’ identification and observation are significant for gaining a deeper understanding of disease pathogenesis and therapeutic mechanisms. Consequently, pinpointing potential miRNA–disease associations (MDA) has become a prominent bioinformatics subject, encouraging several new computational methods given the advances in graph neural networks (GNN). Nevertheless, these existing methods commonly fail to exploit the network nodes’ global feature information, leaving the generation of high-quality embedding representations using graph properties as a critical unsolved issue. Addressing these challenges, we introduce the DAEMDA, a computational method designed to optimize the current models’ efficacy. First, we construct similarity and heterogeneous networks involving miRNAs and diseases, relying on experimentally corroborated miRNA–disease association data and analogous information. Then, a newly-fashioned parallel dual-channel feature encoder, designed to better comprehend the global information within the heterogeneous network and generate varying embedding representations, follows this. Ultimately, employing a neural network classifier, we merge the dual-channel embedding representations and undertake association predictions between miRNA and disease nodes. The experimental results of five-fold cross-validation and case studies of major diseases based on the HMDD v3.2 database show that this method can generate high-quality embedded representations and effectively improve the accuracy of MDA prediction.

## 1. Introduction

MicroRNAs (miRNAs) are a class of small, naturally occurring, non-coding RNA molecules, approximately 21–25 nucleotides in length. Since the end of the last century, when scientist Frank Slack discovered that abnormal miRNA expression in nematodes could lead to problems in tumor biology [1], an increasing number of researchers have focused on the relationship between human miRNA molecules and complex diseases. For example, ectopic expression of miR-196b has been found to increase the spread of leukemia and cause a more rapid onset of leukemia in secondary grafts [2]. Additionally, significant changes in miRNA expression have been observed in patients with major diseases such as breast, gastric, and lung cancers [3,4,5]. As miRNAs continue to prove their importance as biomarkers, they are expected to play an irreplaceable role in cutting-edge medical therapies and become a cornerstone of precision medicine. Therefore, accurately identifying the associations between miRNAs and diseases is of great biological significance.

In recent years, there has been a proliferation of computational-based methods aimed at alleviating the burden on traditional biological experimental researchers and expediting the verification of causal relationships between miRNAs and diseases. These computational methods generally fall into two main categories: feature selection-based methods [6,7,8,9,10] and machine learning-based methods [11,12,13,14,15,16,17,18]. Although both approaches have achieved considerable results in the field of miRNA–disease association prediction, as more MDAs are proven and MDA networks expand, association prediction problems on multidimensional data structures become increasingly complex. Traditional machine learning-based approaches struggle to handle underlying, deep relational networks and uncertain data.

In response to these challenges, researchers have begun to explore the use of deep learning algorithms, such as graph neural networks, which specialize in processing graph data. For example, Ding et al. [19] constructed a variational graph autoencoder to make full use of the known associative graph information based on the representation of heterogeneous networks. Zhang et al. [20] developed a model with a node-level attentional autoencoder, considering the varying importance of different neighboring child nodes to the information of the parent node. Lou et al. [21] proposed the MINIMDA model to improve existing graph convolutional neural networks by displaying aggregated information from higher-order neighborhoods. Tang et al. [22] introduced the MMGCN model, which integrates a multi-source similarity network based on a GCN encoder and a CNN combined decoder to adaptively learn different feature views.

However, existing methods utilize either similar network information or heterogeneous network information. When dealing with similarity features, only local shallow messages are aggregated, ignoring the global node feature information hidden in the network. When utilizing heterogeneous network information, only the association properties of node pairs are considered, neglecting the graph properties embedded in different nodes when encoding the heterogeneous network as a graph. Most importantly, the performance of the model heavily depends on the extent to which the node features are mined and exploited.

In other research domains, researchers [23,24,25] have increasingly shifted their focus towards architectures based on the transformer model to overcome the limitations of graph models when it comes to effectively exploring and learning global information. For instance, Zhang et al. [23] proposed a multi-level transformer-based DTI prediction method to accelerate the screening of effective new drug candidates and enhance the model’s ability to capture complex relationships among multiple types of nodes in complex topologies. Li et al. [24] designed a causal relationship between diseases and genes based on the transformer architecture, which makes better use of multi-source heterogeneous information, and automatically and comprehensively captures the potential multiple interactions between diseases and genes.

These studies demonstrate the feasibility of using transformer-based architectures in their respective domains and show that such models can significantly enhance the feature encoders’ perceptual field. However, none of their studies considered graph properties such as degree centrality and the graph’s shortest path, which are carried by heterogeneous graphs themselves. Inspired by the study of Ying et al. [26], we introduced miRNA–disease heterogeneous network graph properties for the first time in a self-attention-based encoder, aiming to obtain more information-intensive feature embeddings. Based on this, we proposed DAEMDA, a method with a two-channel graph attention mechanism for predicting miRNA–disease associations.

Specifically, we first constructed miRNA and disease similarity networks and miRNA–disease heterogeneous networks under multi-feature graphs. Second, we used graph attention and self-attention-based feature encoders to learn feature information between neighboring nodes and similarity information of the whole graph in parallel, and finally, mature node embedding encodings were obtained. In the end, the node embedding encoding from the dual-channel output is fused using maximum pooling and the association scores between miRNAs and diseases are predicted using a multi-layer perceptron (MLP). To evaluate the performance of our model, we conducted five-fold cross-validation experiments comparing a variety of models with good performance in recent years under mainstream databases and performed ablation experiments and a case study on the model. The results show that our model has an average area under the ROC curve (AUC) of 0.9439 and an area under the precision-recall curve (AUPR) of 0.9429 under the HMDD v3.2 database, which are better than the comparison methods. Our main contributions are as follows:

- The encoder based on the transformer architecture is used to deeply and comprehensively explore the latent node features by fully exploiting the graph properties in the heterogeneous network constructed from multi-feature information so that the node feature embedding is obtained with richer semantic information.
- DAEMDA organically combines node embedding encoding obtained based on graph attention and self-attention encoders to obtain high-quality feature embedding combinations.
- DAEMDA can predict MDA end-to-end, outperforming baseline methods in multiple experiments on publicly available datasets and achieving excellent performance in case studies with more stringent validation criteria.

## 2. Materials and Methods

### 2.1. Experimental Data

#### 2.1.1. Human miRNA–Disease Associations

The Human MicroRNA Disease Database, HMDD (http://www.cuilab.cn/static/hmdd3/data/alldata.xlsx (accessed on 3 August 2023)) [27], provides valuable information regarding the relationships between microRNAs and human diseases, along with relevant references. This information primarily stems from laboratory research findings, encompassing literature-based data mining and human clinical studies. As of now, the database has amassed a total of 35,547 microRNA–disease associations (MDAs) that have been confirmed through experimental papers. In our experiment, to ensure a fair comparison with other models, we opted to work with a benchmark dataset based on the HMDD v3.2 database. This dataset comprises 12,446 associations, involving 853 miRNAs and 591 diseases, all of which we have considered as positive samples. Given the sparsity of positive samples within the entire association graph, we undertook measures to balance the positive and negative samples. We randomly selected an equal number of association data points from samples with known absence of association and those with unknown association status, designating them as negative samples. The combination of these positive and negative samples formed the complete dataset for our experiment. Table 1 presents essential details regarding the dataset employed in this study.

#### 2.1.2. Disease Semantic Similarity

We describe relationships between diseases as directed acyclic graphs (DAGs), represented as $DAG\left(d_{i}\right)=\left(N\;\left(d_{i}\right),\;E\left(d_{i}\right)\right)$, based on MeSH descriptors obtained from the U.S. National Library of Medicine (https://www.ncbi.nlm.nih.gov/ (accessed on 3 August 2023)). In this representation, $N\;\left(d_{i}\right)$ denotes the set of nodes that includes $d_{i}$ itself and has a graph relationship with $d_{i}$, while $E\left(d_{i}\right)$ denotes the set of edges with an ‘is-a’ relationship between those points. Subsequently, we employ the computational method proposed by Wang et al. [28] to calculate the semantic value of each disease. Based on these semantic values, we construct a semantic similarity network for diseases. The disease semantic similarity, denoted as $DSS\left(d_{i},d_{j}\right)$, between any two diseases, ‘$d_{i}$’ and ‘$d_{j}$’, is computed as follows:

(1) $$DSS\left(d_{i},d_{j}\right)=\frac{\sum_{d_{k}\in N\left(d_{i}\right)\cap N\left(d_{j}\right)}\left(D\left(d_{i},d_{k}\right)+D\left(d_{j},d_{k}\right)\right)}{SD\left(d_{i}\right)+SD\left(d_{j}\right)},$$

where $D\left(d_{i},d_{k}\right)$ denotes the semantic score of disease $d_{i},$ and $SD\left(d_{i}\right)$ denotes the semantic value of disease $d_{i}$. The semantic score and semantic value are calculated using Equations (2) and (3), respectively:

(2) $$D\left(d_{i},d_{t}\right)=\{\;\begin{array}{ll} 1, & \mathrm{if}\;d_{i}=d_{t}\\ max\left\{0.5*D\left(d_{i},d_{t}^{\prime }\right)|d_{t}^{\prime }\in children\;of\;d_{i}\right\}, & \mathrm{if}\;d_{i}\neq d_{t}, \end{array}$$

(3) $$SD\left(d_{i}\right)=\sum_{d_{t}\in N\left(d_{i}\right)}D\left(d_{i},d_{t}\right).$$

#### 2.1.3. MiRNA Functional Similarity

According to Wang et al. [28], miRNAs with similar functions are often linked to similar diseases. To facilitate our research, we utilized the MISIM database (http://www.cuilab.cn/files/images/cuilab/misim.zip (accessed on 3 August 2023)), which was developed based on their findings. Our approach involved calculating pairwise miRNA functional similarity matrices, using both the query information from the MISIM database and complementary data from the HMDD v3.2 dataset. This comprehensive data allowed us to construct a miRNA functional similarity matrix, denoted as $MFS\left(m_{i},m_{j}\right)$, where ‘$m_{i}$’ and ‘$m_{j}$’ represent any two miRNAs. The formula for calculating the miRNA functional similarity, $MFS\left(m_{i},m_{j}\right),$ is as follows:

(4) $$MFS\left(m_{i},m_{j}\right)=\frac{\sum_{d\in D\left(m_{i}\right)}\mathrm{DSS}\left(d,d_{j}^{*}\right)+\sum_{d\in D\left(m_{j}\right)}\mathrm{DSS}\left(d,d_{i}^{*}\right)}{\left|D\left(m_{i}\right)\right|+\left|D\left(m_{j}\right)\right|},$$

where $D\left(m_{i}\right)$ denotes the existence of at least one set of diseases related to $m_{i}$, $\left|D\left(m_{i}\right)\right|$ is the number of elements in the set $D\left(m_{i}\right)$, and $d_{j}^{*}$ denotes the disease in $D\left(m_{i}\right)$ that has the greatest semantic similarity to d. Equation (9) is used for calculation:

(5) $$d_{j}^{*}=\underset{d_{j}\in D\left(m_{j}\right)}{argmax}DSS\left(d,d_{j}\right).$$

#### 2.1.4. Gaussian Interaction Profile Kernel Similarity for miRNAs and Diseases

As per the findings of Van Laarhoven et al. [29], our approach is grounded in the miRNA–disease association network. In this network, any disease node can be leveraged to create a similarity network using the GIP kernel similarity. The strength of the GIP kernel similarity between different miRNAs correlates with the similarity of the diseases they are associated with. To facilitate this, we introduce two binary vectors, $IP\left(d_{i}\right)$ and $IP\left(d_{j}\right)$, which characterize the interaction profiles of diseases $d_{i}$ and $d_{j}$. The GIP kernel similarity, denoted as $DGS\left(d_{i},d_{j}\right)$, between diseases $d_{i}$ and $d_{j}$ is calculated as follows:

(6) $$DGS\left(d_{i},d_{j}\right)=exp\left(-\theta_{d}{\left\|IP\left(d_{i}\right)-IP\left(d_{j}\right)\right\|}^{2}\right),$$

the parameter $\theta_{d}$ is employed to regulate the kernel bandwidth, and its definition is presented in Equation (7):

(7) $$\theta_{d}=1/\left(\frac{1}{N_{d}}\sum_{k=1}^{N_{d}}{\left\|IP\left(d_{k}\right)\right\|}^{2}\right),$$

the parameter $N_{d}$ signifies the number of diseases within the miRNA–disease association network. Likewise, we can obtain the GIP kernel similarity $MGS\left(m_{i},m_{j}\right)$ of miRNAs by a similar calculation:

(8) $$MGS\left(m_{i},m_{j}\right)=exp\left(-\theta_{m}{\left\|IP\left(m_{i}\right)-IP\left(m_{j}\right)\right\|}^{2}\right),$$

where the parameter $\theta_{m}$ is defined as shown in Equation (9), and $N_{m}$ denotes the number of miRNAs in the miRNA–disease association network:

(9) $$\theta_{m}=1/\left(\frac{1}{N_{m}}\sum_{k=1}^{N_{m}}{\left\|IP\left(m_{k}\right)\right\|}^{2}\right).$$

#### 2.1.5. Aggregating Similarity Features and Constructing Complex Networks

To better aggregate the neighbor information of miRNA and disease nodes with each other, we constructed miRNA–miRNA homogeneity networks and disease–disease homogeneity networks using known similarity information. Specifically, we first treated the miRNA functional similarity network as an undirected graph and created an M×M binary adjacency matrix, GM, based on the number of miRNAs, M. If there is a functional similarity relationship between miRNA $m_{i}$ and $m_{j}$, then the value of row i and column j of this binary matrix will be marked as 1. Then, we aggregated the miRNA functional similarity matrix and miRNA GIP kernel similarity matrix, and the aggregation result is used as the initial feature matrix of miRNA nodes, so the M-dimensional initial feature vector, $MF_{m\left(i\right)}$, of the miRNA, $m_{i}$, can be expressed as:

(10) $$MF_{m\left(i\right)}=\left(x_{1},x_{2},x_{3},\ldots ,x_{M-1},x_{M}\right),$$

where $x_{1},x_{2},x_{3},\ldots ,x_{M-1},x_{M}$ represents the similarity features of columns 1 to M on row i of the miRNA initial feature matrix, and the similarity features are aggregated as shown in (2):

(11) $$MF\left(m_{i},m_{j}\right)=\{\;\begin{array}{ll} MFS\left(m_{i},m_{j}\right)\; & \mathrm{if}\;MFS\left(m_{i},m_{j}\right)\neq 0\\ MGS\left(m_{i},m_{j}\right) & \mathrm{else} \end{array},$$

where MFS is the miRNA functional similarity matrix, and MGS is the miRNA GIP kernel similarity matrix. After that, we created an N×N binary adjacency matrix, GD, based on the number of diseases, N, and the semantic similarity network of diseases in a similar way to express the correlation between diseases. According to the same method, the initial feature matrix, DF, of the aggregated disease nodes is also created. Finally, we created an M×N binary adjacency matrix, A, based on the known number of miRNAs M and number of diseases, N, in the dataset, and the value of row i and column j of the binary matrix, A, will be marked as 1 if there is a definite association between miRNA $m_{i}$ and disease $d_{j}$. Based on the adjacency matrix, A, we obtained the matrix representation of the miRNA–disease heterogeneous network as follows:

(12) $$G=\left[\begin{array}{cc} 0 & A\\ A^{T} & 0 \end{array}\right]\in {\mathbb{R}}^{\left(M+N\right)\times \left(M+N\right)}.$$

### 2.2. Model Framework

**Figure 1:**
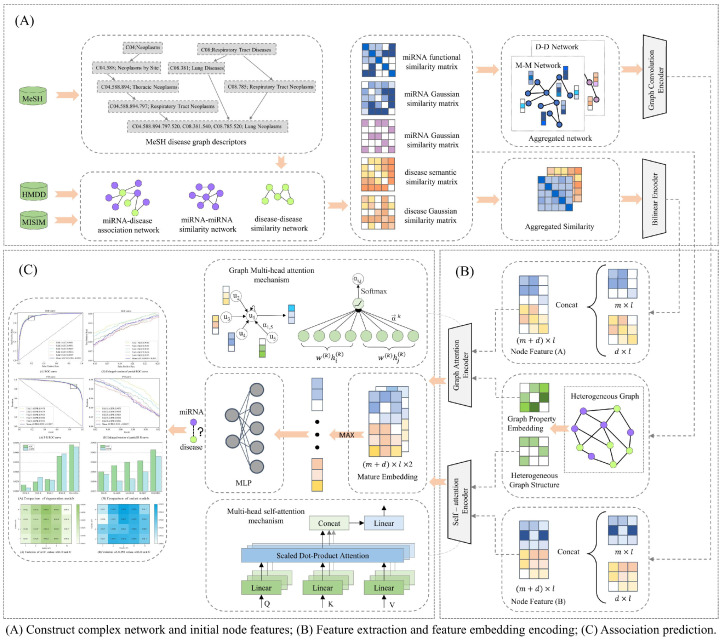
The overall structure of DAEMDA.

To better aid experimental researchers in the discovery of new biomarkers and achieve more efficient and accurate predictions of miRNA–disease associations, we introduce a computational method named DAEMDA, outlined in Figure 1. This method comprises three primary steps: (I) Construction of complex networks and initial features of nodes: generate interaction networks and original feature matrices based on similarity and correlation information provided by experimental materials. (II) Feature extraction and feature embedding encoding: mining the information of nodes and heterogeneous graphs using two attention encoders, which are used to generate high-quality feature embedding encoding. (III) Association prediction: the two-channel feature embedding encodings are fused, and the final prediction results are obtained using a neural network classifier.

#### 2.2.1. Graph Attention-Based Encoder

A graph attention network (GAT) combines the feature encoding of nodes based on the topological relationships between the nodes of a heterogeneous graph. In simple terms, GAT selects the relevant information of its neighboring nodes by the attention mechanism for each node and weighted summation to form a summarized feature representation. This feature representation not only includes the node’s own feature information but also considers the relevant information of its neighboring nodes, which is equivalent to compressing the information of the whole heterogeneous graph into a global feature vector.

The GAT encoder takes the heterogeneous graph, $G=\left(V,E\right)\in {\mathbb{R}}^{\left(M+N\right)\times \left(M+N\right)}$, as input, where E is the set of edges, V is the set of nodes, and for any node, $V_{i}$, has its feature vector, ${\vec{h}}_{v_{i}}\in {\mathbb{R}}^{l}$, where l is the dimension of the feature vector, and in this study, the feature vectors of nodes are obtained using the node features with information about the shallow neighbors of the nodes after encoding the similarity network through the GCN encoder. For any $MDA\left(V_{i},V_{j}\right)\in E$, node $V_{i}$ and its neighbor node, $V_{j}\in N\left(i\right)$, where N(i) denotes the set of neighbor nodes of $V_{i}$. Then, the similarity coefficient, $e_{ij}$, between nodes $V_{i}$ and $V_{j}$ is defined as follows:

(13) $$e_{ij}=\sigma \left({\vec{a}}^{T}\left[\vec{W}{\vec{h}}_{v_{i}}∥\vec{W}{\vec{h}}_{v_{j}}\right]\right),$$

where ∥ denotes the vector connection operation, $\vec{a}$ is a self-learning parameter used to control the importance weights of interactions between nodes, $\vec{W}$ is a feature transformation parameter that can be learned, and σ is the LekyReLU activation function. For the central node, $V_{i}$, after obtaining the importance among all its neighboring nodes, the softmax function is applied to normalize all $e_{ij}$ to obtain the attention coefficients, $a_{ij}$. Then, for the node $V_{i}$, we weight and sum its feature vector, ${\vec{h}}_{v_{i}},$ with the corresponding attention coefficient, $a_{ij}$, between its neighboring nodes to obtain a weighted sum vector for node $V_{i}$:

(14) $$z_{i}=\sum_{j\in N\left(i\right)}a_{ij}\vec{W}{\vec{h}}_{v_{j}}.$$

In order to improve the expressiveness and accuracy of GAT and to be able to capture the features of multiple information domains at the same time, we extend the calculation of attention coefficients of nodes in the GAT model to multiple heads, and finally, we stitch the weights and vectors of multiple heads together and perform nonlinear activation to obtain the final node feature vector, $Z_{i}^{\prime }$, of node $v_{i}$:

(15) $$Z_{i}^{\prime }=\overset{k}{\underset{k=1}{∥}}\sigma \left(\sum_{j\in N_{i}}\alpha_{ij\;}^{\left(k\right)}\;\;{\vec{W}}^{\left(k\right)}{\vec{h}}_{v_{j}}^{\left(k\right)}\right),$$

where *K* represents the number of multi-headed attention heads. Also, to improve the fusion of feature information, we apply a jumping knowledge module to merge the output of each layer in the multi-layer GAT. In the end, the merged vectors are linearly transformed to obtain the l-dimensional feature embedding generated by the GAT encoder:

(16) $$H_{GAT}=Linear\left(concat\left(GAT_{1},\ldots ,GAT_{n-1},GAT_{n}\right)\right),$$

where $GAT_{i}$ represents the feature embedding matrix of the layer i GAT output, and n is the total number of GAT layers used in this encoder.

#### 2.2.2. Self-Attention-Based Encoder

Here, we designed the transformer global feature encoder for this method following an architectural pattern similar to the transformer encoder studied by Vaswani et al. [30]. The transformer encoder in our study is a serial combination of several identical layers, and each individual layer contains two separate sub-layers, a multi-head attention layer, and a feedforward neural network layer. In contrast to the original method, instead of using the results of the node features after the masking operations using positional coding as the input to the encoder, we obtained the input to the encoder according to the following method:

(17) $$F_{md}=concat\left(Linear\left(MF\right),Linear\left(DF\right)\right)+H_{\deg\left(G\right)},$$

where MF and DF are the similarity feature matrices of the network nodes, and $H_{\deg\left(G\right)}$ are learnable matrices generated based on degree centrality, which reflects the important status of miRNA and disease nodes in the network, and introducing degree centrality matrices can make better use of graph properties to make the model more easily focus on nodes with high contributions. Considering the superiority of the self-attention mechanism in global structure learning, each node can learn the information of any position, but this will cause the loss of the associativity information of the nodes in the graph. The association between nodes in a multidimensional space can be defined by connectivity, which we use to learn our defined matrix, $B_{\varphi \left(G\right)},$ by measuring the spatial relationship between two nodes in the graph, i.e., the shortest path between two nodes. By introducing this matrix as a bias quantity into the self-attention operation, we can make our self-attention encoder focus more on the nodes close to it and less on the nodes far from it when encoding the nodes. In summary, our self-attention encoder is described as follows:

(18) $$\{\begin{array}{l} Q=F_{md}\times W_{q}^{\;}\\ K=F_{md}\times W_{k}^{\;}\\ V=F_{md}\times W_{v}^{\;} \end{array},$$

(19) $$Attention=softmax\left(\frac{QK^{T}}{\sqrt{d}}+B_{\varphi \left(G\right)}\;\right)V,$$

where $F_{md}\in {\mathbb{R}}^{\left(M+N\right)\times k}$ is the input to the self-attention encoder, W is the parameter matrix that is used for the linear transformation when performing the self-attention operation, and the result of the linear transformation $\left(Q,K,V\right)\in {\mathbb{R}}^{\left(M+N\right)\times d}$, d, is the dimension of the matrices Q, K, and V. Suppose we arrange the h self-attention layers in parallel into a multi-head attention layer, the output matrix can be written as:

(20) $$MultiHead{\left(F_{md}\right)}^{\left(h\right)}=concat\left(head_{1},\ldots ,head_{h-1},head_{h}\right)W_{O},$$

(21) $$head_{i}=Attention\left(F_{md}\times W_{q}^{i},F_{md}\times W_{k}^{i},F_{md}\times W_{v}^{i}\right),i=1,\ldots ,h.$$

After this, in order to make the deep network more stable, converge faster, and effectively eliminate the gradient disappearance and gradient explosion problems, we also layer-normalized the output matrix of the multi-head attention layer and finally obtained the feature encoding matrix, $H_{Encode}$, as follows:

(22) $$H_{Encode}=FNN\left(LN\left(MultiHead{\left(F_{md}\right)}^{\left(h\right)}\right)\right),$$

where LN is layer normalization, and FNN is a feedforward neural network composed of two linear transformations and one nonlinear activation (using the ReLU activation function) together. The addition of feedforward neural network layers can enhance the expressiveness of the model to a great extent, improve the generalization ability, and decouple the importance hidden by the feature representation at different locations.

#### 2.2.3. Predicting miRNA–Disease Associations

With the two feature encoders, we obtained the feature embedding matrices for the two-channel output. In order to highlight the important features and improve the sensitivity of the model to important information, we performed a maximum pooling operation for these. Simply speaking, the maximum value of the corresponding positions of the two matrix species is selected as the pooling result. This operation can keep the stronger features and ignore those relatively weaker ones, and, finally, we obtain the prediction result of the model:

(23) $$A^{\prime }=maxpooling\left(H_{GAT}∥H_{Encode}\right).$$

In order to enable the model optimization, minimize the difference between the true and predicted associations, and thus improve the prediction accuracy of the model, we use the loss function of cross-entropy:

(24) $$L\left(y,p\right)=-\frac{1}{N}\sum_{i=1}^{N}\left[y_{i}log\left(p_{i}\right)+\left(1-y_{i}\right)log\left(1-p_{i}\right)\right],$$

where y is the true label vector, p is the label vector predicted by the model, and N is the number of samples.

## 3. Results

### 3.1. Experimental Settings

#### 3.1.1. Parameter Settings

The selection of hyperparameters holds immense significance in the realm of deep learning models. Optimal hyperparameter choices can lead to improvements in training speed, a reduction in overfitting, and ultimately result in enhanced performance. In our pursuit of the best-performing hyperparameters, we conducted a series of experiments. Finally, we set the number of heads in the multi-head attention layer to four, set the length of the shortest path to eight, set the number of neurons in the hidden layer of the feedforward neural network to 2048, and set the dimensionality of feature embedding to 512 dimensions. In order to reduce overfitting and improve the generalization ability of the model, we use the Dropout technique to randomly remove neurons from the model and set the Dropout rate = 0.5. In addition, we use the Adam optimizer to update the weights of the network and set the learning rate to 1 × 10^−4^ to better reduce the loss of the model.

#### 3.1.2. Baselines

We selected six high-performing methods, as shown below, as baseline methods to compare with our prediction methods, and the parameters of all the compared methods were based on the best parameters of each reported in the original study.

- NIMGSA [31]: A neural inductive matrix completion-based method with graph autoencoders and self-attention mechanism for miRNA–disease association prediction.
- AGAEMD [20]: The authors considered the node-to-node attention profile in the heterogeneity graph and dynamically updated the miRNA functional similarity matrix during model iterations.
- ERMDA [32]: The authors utilize a resampling method to process the existing data and use integrated learning to introduce a soft voting method for the final prediction of the association.
- GATMDA [33]: A new computational method to discover unknown miRNA–disease associations based on a graph attention network with multi-source information, which effectively fuses linear and non-linear features.
- SFGAE [34]: Association prediction between two classes of nodes was accomplished by constructing miRNA self-embeddings and disease self-embeddings, independent of graph interactions between the two classes of graphs.
- AMHMDA [35]: GCN is used to extract multi-perspective node information from multi-similarity network species for constructing hypergraphs, and an attention mechanism is used to fuse features from hypergraph nodes for predicting MDA.

#### 3.1.3. Experimental Environment

##### Hardware Equipment Used in This Study

- CPU: Intel Xeon Platinum 8255C, 2.50 GHz;
- GPU: RTX 2080Ti (11 GB), cuda12.0;
- Memory: 40 GB.

This study is based on the Ubuntu 20.04 operating system, Python 3.8 environment, using the Pytorch framework to implement the model and complete the experiments.

#### 3.1.4. Evaluation Metrics

We mainly used four commonly used evaluation metrics, namely, an area under the ROC curve (AUC), an area under the exact recall curve (AUPR), accuracy (ACC), F1 score (F1), precision, and recall rate to evaluate the method performance. Among them, AUC is based on the true positive rate, $=\frac{TP}{TP+FN}$, and false-positive rate, $=\frac{FP}{TN+FP}$, which reflects the relationship between sensitivity and the specificity of the prediction model, and AUPR is based on the precision, $=\frac{TP}{TP+FP},$ and recall rate, $=\frac{TP}{TP+FN}$. The AUC and AUPR values are two important metrics to evaluate the prediction effectiveness, respectively; in short, the larger the AUC and AUPR the better the performance of the model. The remaining metrics are defined as shown below:

(25) $$ACC=\frac{TP+TN}{TP+TN+FP+FN}$$

(26) $$F1=2\cdot \frac{precision\cdot recall}{precision+recall}$$

### 3.2. Performance Evaluation

To conduct a thorough and unbiased evaluation of our method, we employed a five-fold cross-validation (5-CV) approach on the HMDD v3.2 dataset. Within the 5-CV experiments, we executed five distinct operations with our prediction model: (1) The entire dataset was initially and randomly partitioned into five equally sized subsets. (2) During each cross-validation iteration, four of these subsets were utilized for training. One subset was designated for validation. It is essential to emphasize that each subset was exclusively employed as validation data once throughout the entire 5-CV process. The following figures and table show the results of our experiments.

**Figure 2:**
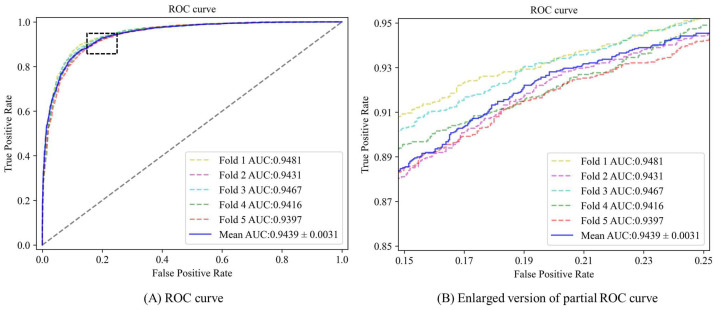
ROC curve for DAEMDA on HMDD v3.2 dataset by 5-CV experiment.

**Figure 3:**
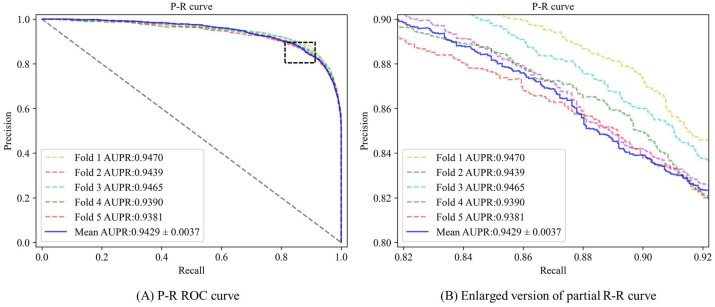
P-R curve for DAEMDA on HMDD v3.2 dataset by 5-CV experiment.

From the experimental results presented in Figure 2 and Figure 3, it is evident that the AUC (area under the ROC curve) values of the five-fold cross-validation model are as follows: 0.9481, 0.9431, 0.9467, 0.9416, and 0.9397, respectively. Additionally, the area under the Precision-Recall curve (AUPR) values are observed to be: 0.9470, 0.9439, 0.9465, 0.9390, and 0.9381. Combined with the experimental data in Table 2, in this experiment, the mean AUC value of DAEMDA under the HMDD v3.2 dataset is 0.9439, the mean AUPR value is 0.9429, the mean accuracy value is 0.8744, the mean F1 score is 0.8746, the mean precision value is 0.8747, and the mean recall is 0.8763. The corresponding standard deviations are 0.0031, 0.0037, 0.0057, 0.0048, 0.0261, and 0.0286, respectively. Based on these comprehensive experimental results obtained from our study design, we can assert with confidence that our model exhibits exceptional stability in its performance characteristics. This remarkable stability can likely be attributed to the effective incorporation of normalization techniques within our self-attention-based encoder architecture.

In order to comprehensively evaluate the performance of the model in real applications and assess its generalization ability, we adopted a more rigorous testing method. Specifically, we used the HMDD v3.2 dataset, which was divided into a training set and an independent test set (with 4978 positive and negative samples) according to the ratio of 80%-20%. In order to simulate a real scenario where associated data are often missing, we performed five-fold cross-validation on the training set, using only 64% of the entire dataset for training. Subsequently, we selected the best-performing models from the cross-validation phase and conducted experiments on an independent test set. The confusion matrix of the experimental results is shown in Table 3.

Further analysis of the data within the confusion matrix resulted in the following performance metrics for the model on the independent test set: an AUC value of 0.9403, an AUPR value of 0.9381, an accuracy value of 0.8714, an F1 value of 0.8724, a precision value of 0.8661, and a recall value of 0.8787. Although the model’s performance on the independent test set is slightly lower than that on the validation set, the overall performance remains at a high level. This observation underscores the robustness of our model.

**Figure 4:**
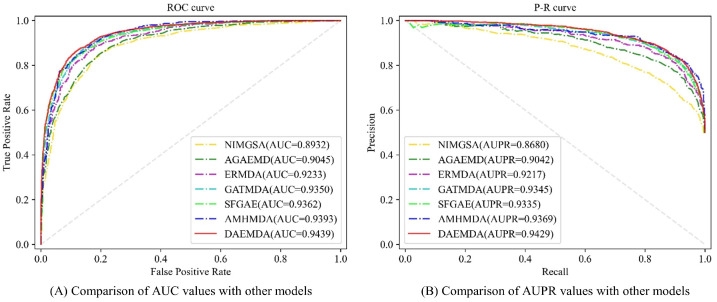
ROC and PR curves of different methods.

To better compare our model with other methods scientifically and fairly, we conducted a five-fold cross-validation-based experimental comparison with the baseline method using AUC and AUPR as rating metrics under the HMDD v3.2 dataset, and the comparison results are shown in Figure 4 and Table 4. It can be seen that the performance of the model is superior to the comparison methods in all the cases.

### 3.3. Ablation Experiment

To verify the effectiveness of generating feature embeddings in our model using a dual-channel mixture, we conducted ablation experiments on the HMDD v3.2 dataset. Specifically, we constructed four variants of DAEMDA: DAE-A indicates that our model uses the self-attention encoder without adding graph attribute information, DAE-B indicates that our model uses the traditional graph attention-based encoder, DAE-C indicates that our model does not use the self-attention-based encoder, and DAE-D indicates that our model does not use the graph attention-based encoder. The experimental results are shown in Table 5.

Meanwhile, in order to obtain the best performance of the model, we designed four variants for the combination of the two-channel feature embedding, namely, the linear change-based combination, M-LIN, the dot product-based combination, M-DOT, the mean pooling-based combination, M-MEAN, and the summation-based combination, M-ADD. The experimental results on the HMDD v3.2 dataset are shown in Table 6.

**Figure 5:**
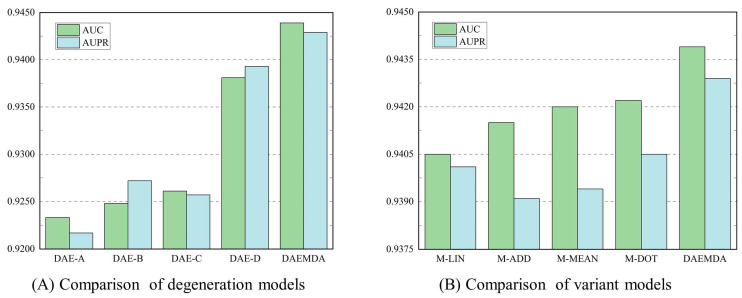
Results of ablation experiments on the HMDD v3.2 dataset.

We can see from the experimental results in Table 6 and Figure 5 that the best performance is obtained with the maximum pooling-based combination used in this method. Based on the above two sets of experiments, we can conclude that (1) both of our encoders are effective, and the absence of either encoder in our model causes a loss in performance; (2) our improved approach is effective, and our encoders perform better compared to using traditional encoders; (3) the way the features are combined is also important to some extent, and choosing the right combination gives the model the relatively best performance.

### 3.4. Parameter Analysis

#### 3.4.1. Number of Attention Heads

**Figure 6:**
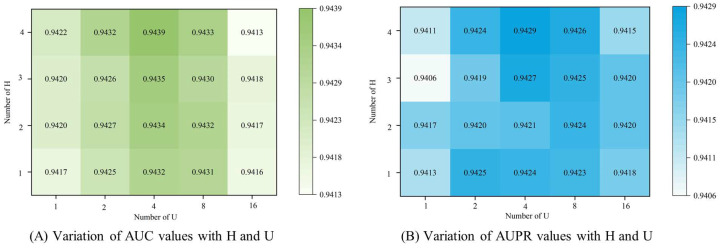
The influence of the number of attention heads on the experimental results.

Choosing an appropriate number of multi-head attention heads can improve the performance of the model. If too few heads are chosen, important information may be lost when learning features, while choosing too many heads can increase the complexity of the model or even learn invalid information. We compare the performance of the model with different numbers of self-attention encoder heads, U, and graph attention encoder heads, H, through several experiments. The results of the experiments are shown in Figure 6, which shows that the performance of the model is relatively optimal when U = 4 and H = 4, and the model is more sensitive to the change of U than to the change of H.

#### 3.4.2. Number of Feature Dimension

**Figure 7:**
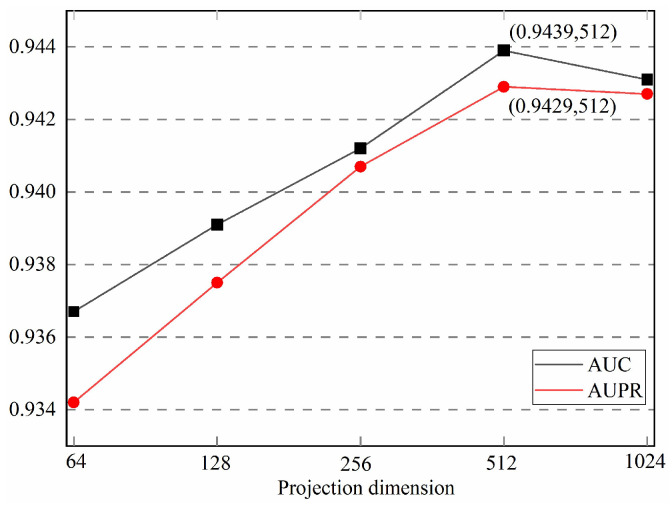
The influence of the number of feature encoding dimensions on the experimental results.

The size of the feature dimension encoded by the encoder in deep learning is also one of the important factors affecting the performance of the model. Generally speaking, the larger the feature dimension, the more information the model has, but it also increases the computational burden and the risk of overfitting. While a lower feature dimension can reduce the computational burden, it also brings problems such as information loss and underfitting. Therefore, a reasonable choice of feature dimension size is crucial to the performance of the model. So, in order to obtain the optimal feature encoding dimension, we conducted experiments on different feature dimensions. The experimental results are shown in Figure 7, and we can see that the model has the best performance when the feature dimension is 512.

## 4. Case Study

**Figure 8:**
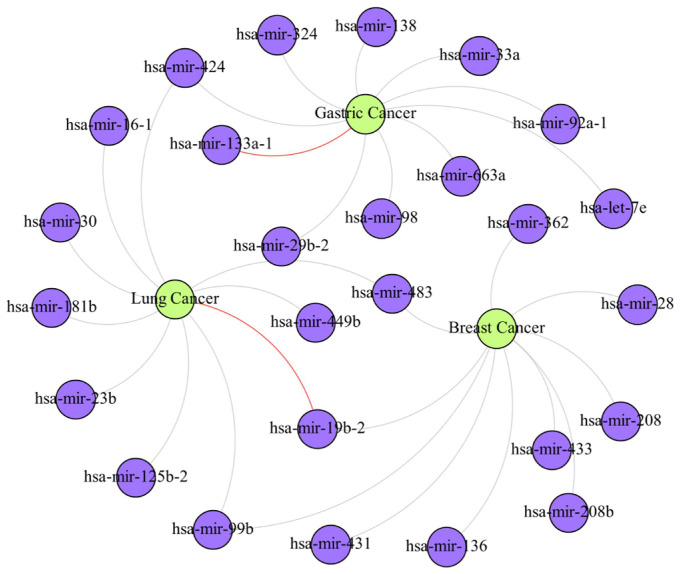
The miRNA–disease association sub-network obtained through experiments. In the figure, purple nodes represent miRNAs, green nodes represent diseases, gray edges between the two types of nodes represent the associations that have been confirmed by databases, and red edges represent associations predicted by the present method that have a high probability of correlation but have not yet been confirmed.

Cancer is a common and serious disease, and many studies in recent years have shown that specific miRNA expression changes are associated with cancer progression, metastasis, and prognosis. To further verify the validity of our proposed model, we conducted case studies on three different cancers. Specifically, we trained our model based on the data from HMDD v3.2 and removed the association data associated with the above diseases in the association network sequentially. Then, we ranked all the miRNAs predicted for the candidate diseases according to their prediction scores, and we validated the ranked results using the HMDD v3.2 database. The top 30 miRNAs predicted for the three diseases were all validated by the HMDD v3.2 database, after which we again selected the top 10 miRNAs among the top 100 miRNAs, with positive prediction results that were not validated by the HMDD database, and validated them using the dbDEMC 3.0 database (https://www.biosino.org/dbDEMC/index (accessed on 3 August 2023)) [36]. The prediction results are shown in Table 7 and Figure 8. By this more rigorous validation than that of previous researchers, among the top 10 miRNAs regarding breast cancer association prediction, all could be validated by the dbDEMC 3.0 database. Among the top 10 miRNAs regarding gastric cancer association prediction, except for hsa-mir-133a-1, which was not supported by relevant literature, the remaining 9 miRNAs were all validated in dbDEMC 3.0. Among the top 10 miRNAs regarding lung cancer association prediction, the overall prediction accuracy reached 90%, except for hsa-mir-19b-2, which was not supported by relevant literature.

## 5. Discussion

Our study differs from previous studies in that, first, instead of directly considering local feature information between nodes and neighboring nodes, or nodes and meta-paths in the graph, we integrate the global feature information of the nodes in the network. It also integrates the graph properties of the network when mining the global information features, which enables the model to discover high-quality inner information more efficiently and generate embedding representations that are more favorable for MDA prediction. Furthermore, we refrain from directly employing GCN to aggregate neighboring node features. Instead, we opt to re-encode the shallow feature information after GCN encoding, using node-level attention. This enhances the aggregation of inter-node correlation information and network topology within complex networks. In addition, we have designed an embedding combination method tailored to this model, enabling the organic fusion of the dual-channel feature embeddings obtained from the feature encoder.

Our method excels at concentrating on the global feature information of nodes, effectively leveraging the graph properties to create high-quality embeddings, bolstering the model’s capacity to learn node representations, and generating superior embedded representations based on both self-attention and graph attention mechanisms. Moreover, DAEMDA demonstrates outstanding predictive performance in comparative experiments, cross-validation, and case study experiments. Therefore, DAEMDA serves as an effective and invaluable research tool for guiding and advancing research in the field of microRNAs.

However, the biological process of miRNAs involves multiple segments; products of different stages in the same miRNA can play different roles in targeting different target genes or in different biological processes, and this is likely to create variability in the association between miRNAs and different diseases. Studies [37,38], for example, investigated the effects of different end products of miR-143 on hepatocellular carcinoma and colon cancer, respectively. In the future, as more mature miRNA information is refined, in order to predict the association between relevant miRNAs and diseases at a finer granularity, we will expand the training data and combine more biological characterization information to achieve this purpose. This will help us to understand the intricate relationship between miRNAs and diseases in a more detailed way, and thus promote the progress of related fields.

## Figures and Tables

**Table 1:** Basic characteristics of the HMDD v3.2 dataset. # nodes denotes the number of nodes; # edges denotes the number of edges; # density denotes the density of the graph; # degree denotes the average degree; # Ave_cen denotes the point degree centrality.

Property	Full Data	miRNA	Disease
# nodes	1444	853	591
# edges	12,446	-	-
# density	0.0247	-	-
# degree	17.238	14.591	21.059
# Ave_cen	0.0119	0.0101	0.0146

**Table 2:** Results of prediction performance for DAEMDA on HMDD v3.2 dataset by 5-CV experiment.

Testing Set	Accuracy	F1-Score	Precision	Recall
1	0.8737	0.8682	0.9076	0.8321
2	0.8777	0.8763	0.8862	0.8666
3	0.8797	0.8817	0.8671	0.8967
4	0.8773	0.8763	0.8830	0.8698
5	0.8638	0.8706	0.8294	0.9160
Mean	0.8744 ± 0.0057	0.8746 ± 0.0048	0.8747 ± 0.0261	0.8763 ± 0.0286

**Table 3:** The confusion matrix obtained by experiments on independent test sets.

True Labels	Predicted Labels	
	Yes MDA	No MDA
Yes MDA	TP = 2187	FN = 338
No MDA	FP = 302	TN = 2151

**Table 4:** Comparison with other methods on HMDD v3.2 dataset.

Method	AUC	AUPR
NIMGSA	0.8932	0.8680
AGAEMD	0.9045	0.9042
ERMDA	0.9233	0.9217
GATMDA	0.9350	0.9345
SFGAE	0.9362	0.9335
AMHMDA	0.9393	0.9369
DAEMDA	0.9439	0.9429

**Table 5:** The comparison results of DAEMDA and its degeneration models.

Method	DAE-A	DAE-B	DAE-C	DAE-D	DAEMDA
AUC	0.9233	0.9248	0.9261	0.9381	0.9439
AUPR	0.9217	0.9272	0.9257	0.9393	0.9429

**Table 6:** The comparison results of DAEMDA and its variant models.

Method	M-LIN	M-DOT	M-MEAN	M-ADD	DAEMDA
AUC	0.9405	0.9415	0.9420	0.9422	0.9439
AUPR	0.9401	0.9391	0.9394	0.9405	0.9429

**Table 7:** Top 10 predicted miRNAs associated with breast cancer, gastric cancer, and lung cancer.

Cancer	Top 10 Prediction					
	Rank	miRNA	Evidence	Rank	miRNA	Evidence
Breast Cancer	1	hsa-mir-28	dbDEMC	6	hsa-mir-362	dbDEMC
	2	hsa-mir-483	dbDEMC	7	hsa-mir-208	dbDEMC
	3	hsa-mir-99b	dbDEMC	8	hsa-mir-19b-2	dbDEMC
	4	hsa-mir-136	dbDEMC	9	hsa-mir-433	dbDEMC
	5	hsa-mir-431	dbDEMC	10	hsa-mir-208b	dbDEMC
Gastric Cancer	1	hsa-mir-29b-2	dbDEMC	6	hsa-mir-92a-1	dbDEMC
	2	hsa-let-7e	dbDEMC	7	hsa-mir-98	dbDEMC
	3	hsa-mir-33a	dbDEMC	8	hsa-mir-324	dbDEMC
	4	hsa-mir-424	dbDEMC	9	hsa-mir-138	dbDEMC
	5	hsa-mir-133a-1	Unconfirmed	10	hsa-mir-663a	dbDEMC
Lung Cancer	1	hsa-mir-424	dbDEMC	6	hsa-mir-99b	dbDEMC
	2	hsa-mir-125b-2	dbDEMC	7	hsa-mir-30	dbDEMC
	3	hsa-mir-181b	dbDEMC	8	hsa-mir-483	dbDEMC
	4	hsa-mir-23b	dbDEMC	9	hsa-mir-449b	dbDEMC
	5	hsa-mir-19b-2	Unconfirmed	10	hsa-mir-16-1	dbDEMC

## Data Availability

The source code of our model is available at https://github.com/Linda908/DAEMDA (accessed on 3 August 2023).
